# Declining eGFR and Uremia Are Associated with Remnant Cholesterol Accumulation and Reduced HDL-C in Non-Dialysis Chronic Kidney Disease

**DOI:** 10.3390/jcm15082918

**Published:** 2026-04-11

**Authors:** Hanan Alyami, Fahd A. Alshuweishi, Nadiah A. Baghdadi, Meshal Marzoog Al-Sharafa, Ali Jaber Alqahtani, Yazeed Alshuweishi

**Affiliations:** 1 Department of Medical and Surgical Nursing, College of Nursing, Princess Norah Bint Abdurrahman University, Riyadh 11564, Saudi Arabia; hmalyami@pnu.edu.sa; 2 King Fahad Kidney Center, King Saud Medical City, Riyadh 12746, Saudi Arabia; falshuweishi@ksmc.med.sa; 3 Department of Nursing Management and Education, College of Nursing, Princess Nourah bint Abdulrahman University, Riyadh 11671, Saudi Arabia; nabaghdadi@pnu.edu.sa; 4 Tissue Banking Section, Research Department, Health Science Research Center, Princess Nourah bint Abdulrahman University, Riyadh 84428, Saudi Arabia; mmalsharafa@pnu.edu.sa; 5 Department of Clinical Laboratory Sciences, College of Applied Medical Sciences, King Saud University, Riyadh 12372, Saudi Arabia; 6 College of Medical and Health Sciences, Liwa University, Abu Dhabi 41009, United Arab Emirates; ali.alqahtani@lu.ac.ae; 7 Center of Excellence in Biotechnology Research (CEBR), King Saud University, Riyadh 12372, Saudi Arabia

**Keywords:** chronic kidney disease, remnant cholesterol, HDL-C, dyslipidemia, uremia, eGFR

## Abstract

**Background:** Cardiovascular risk in chronic kidney disease (CKD) remains high despite frequently normal conventional lipid parameters. The extent to which lipid patterns vary across CKD severity and metabolic complications remains incompletely characterized. Therefore, this study evaluated lipid patterns and their associations with renal function and CKD-related metabolic complications. **Methods:** This retrospective cross-sectional study included 229 CKD patients from the nephrology clinic at King Saud Medical City. Single-time-point laboratory data and clinical variables were extracted from medical records. Patients were stratified by KDIGO eGFR stage, uremia, and phosphate status. Lipid parameters were analyzed using nonparametric tests, multivariable regression, and ROC analysis. **Results:** Among 229 CKD patients, the most prevalent lipid abnormalities were low HDL-C (49.8%) and elevated remnant cholesterol (RC) (31.4%). HDL-C was reduced and RC increased with declining eGFR, while TC, LDL-C, and TG remained unchanged. In uremia, HDL-C remained reduced and RC increased, with additional reductions in TC and LDL-C, whereas TG did not differ. No significant lipid changes were observed with hyperphosphatemia. In multivariable analyses, HDL-C was positively associated with eGFR (β = 48.8, *q* = 0.003) and inversely associated with BUN (β = −14.3, *q* = 0.0014), while RC showed an inverse association with eGFR (β = −19.9, *q* = 0.0005) and a positive association with BUN (β = 3.37, *q* = 0.0315). These relationships remained independent of age, sex, BMI, smoking status, hypertension, and type 2 diabetes. ROC analysis further demonstrated the moderate discriminatory ability of HDL-C and RC for identifying CKD stages and uremia. **Conclusions:** Alterations in HDL-C and RC were independently associated with renal function and uremic status. These findings suggest that lipoprotein composition may reflect metabolic disturbances accompanying CKD progression.

## 1. Introduction

Chronic kidney disease (CKD) is a major global health burden defined as abnormalities of kidney structure or function lasting at least three months, including a decreased glomerular filtration rate (eGFR < 60 mL/min/1.73 m^2^) or markers of kidney damage such as albuminuria [1]. According to the Global Burden of Disease 2023 analysis, approximately 788 million adults worldwide were living with CKD in 2023, with an age-standardized prevalence of around 14.2% [2]. In Saudi Arabia, a recent nationwide cross-sectional study reported a CKD prevalence of approximately 4.76% in the general population [3].

Importantly, CKD is strongly associated with an increased risk of cardiovascular disease (CVD), which remains the leading cause of morbidity and mortality among affected individuals [4]. Notably, cardiovascular risk is already substantially elevated in the earlier stages of CKD, including among patients who have not yet progressed to dialysis, emphasizing the need to identify metabolic and biochemical alterations that contribute to this excess risk [5,6]. Evidence indicates that statin therapy can reduce cardiovascular events in patients with pre–end-stage CKD as well as in kidney transplant recipients; however, such benefits are not consistently observed in patients receiving dialysis, highlighting the distinct pathophysiology of advanced CKD [7]. This discrepancy suggests that conventional lipid-lowering strategies primarily targeting LDL-C may be less effective in advanced disease, and underscores the importance of focusing on non-dialysis CKD patients, where early intervention may still modify cardiovascular risk. Moreover, it points to the need to better characterize non-traditional lipoprotein abnormalities, as statins may not adequately address other atherogenic particles such as remnant lipoproteins.

Dyslipidemia is a well-recognized yet complex contributor to atherosclerotic cardiovascular disease in CKD [8]. However, lipid abnormalities in CKD differ from the classical dyslipidemia observed in the general population. While hypertriglyceridemia and reduced high-density lipoprotein cholesterol (HDL-C) are frequently observed, low-density lipoprotein cholesterol (LDL-C) and total cholesterol (TC) often remain within normal ranges, particularly in non-dialysis CKD patients [9]. This atypical lipid profile partly explains why conventional lipid markers may underestimate atherogenic risk in CKD patients and why lipid-lowering interventions have shown heterogeneous cardiovascular benefits across CKD stages [10]. Consequently, there is increasing interest in alternative lipid measures that may better reflect atherogenic burden in CKD. In recent years, attention has shifted toward alternative lipid measures that may better capture atherogenic burden in CKD. Remnant cholesterol (RC), which represents cholesterol carried in triglyceride-rich lipoprotein remnants such as very-low-density lipoproteins (VLDL) and intermediate-density lipoproteins (IDL), has emerged as a potentially important marker of residual cardiovascular risk [11,12]. RC reflects impaired clearance of triglyceride-rich particles, a metabolic disturbance that is particularly relevant in CKD due to reduced lipoprotein lipase activity, chronic inflammation, and altered hepatic lipid handling [13]. Moreover, non–high-density lipoprotein cholesterol (non-HDL-C) is defined as total cholesterol minus HDL-C and represents the cholesterol content of all atherogenic lipoproteins, including LDL, very-low-density lipoproteins (VLDL), intermediate-density lipoproteins (IDL), lipoprotein(a), and remnant lipoproteins [14]. It has been shown to be a strong predictor of cardiovascular risk across diverse populations [15,16,17]. Importantly, RC may increase even when LDL-C and non–HDL-C remain unchanged, suggesting a redistribution of cholesterol among lipoprotein fractions rather than a global increase in atherogenic cholesterol [11,18,19].

Beyond declining glomerular filtration rate, CKD is characterized by metabolic disturbances related to uremic toxin accumulation and disorders of mineral metabolism [20,21]. Elevated urea levels reflect impaired renal clearance and are associated with systemic inflammation, oxidative stress, and protein-energy wasting, all of which may influence lipid metabolism [22,23,24]. Similarly, hyperphosphatemia, a hallmark of advanced CKD and CKD-mineral and bone disorder (CKD-MBD), has been linked to vascular calcification and adverse cardiovascular outcomes [20,25]. However, whether urea accumulation or phosphate disturbances are associated with lipid abnormalities in CKD remains incompletely understood, and their relationship with dyslipidemia may differ from that of traditional metabolic risk factors.

Despite increasing recognition of remnant cholesterol and non-HDL-C as clinically relevant lipid markers, data evaluating their distribution across CKD stages, particularly among non-dialysis CKD patients, remain limited. Furthermore, few studies have examined whether lipid abnormalities in CKD are primarily driven by declining renal function itself or by metabolic disturbances such as uremia and phosphate imbalance. Addressing these gaps is important for improving cardiovascular risk stratification in CKD populations. Therefore, the present study aimed to evaluate patterns of lipid parameters, including TC, LDL-C, HDL-C, TG, non–HDL-C, and RC across KDIGO-defined eGFR stages in non-dialysis CKD patients. In addition, the study examined differences in lipid profiles according to uremic status and phosphate status within the overall cohort, with particular emphasis on determining whether uremia and hyperphosphatemia are independently associated with lipid abnormalities after accounting for CKD severity and relevant clinical covariates.

## 2. Methods

### 2.1. Study Design and Population

This retrospective observational study was conducted using patient records retrieved from the Nephrology Clinic at King Saud Medical City, Riyadh, Saudi Arabia. The study included adult patients diagnosed with chronic kidney disease (CKD) who attended the nephrology clinic between January 2021 and December 2023 and underwent routine laboratory evaluation during their clinical visits.

### 2.2. Inclusion and Exclusion Criteria

**Figure 1:**
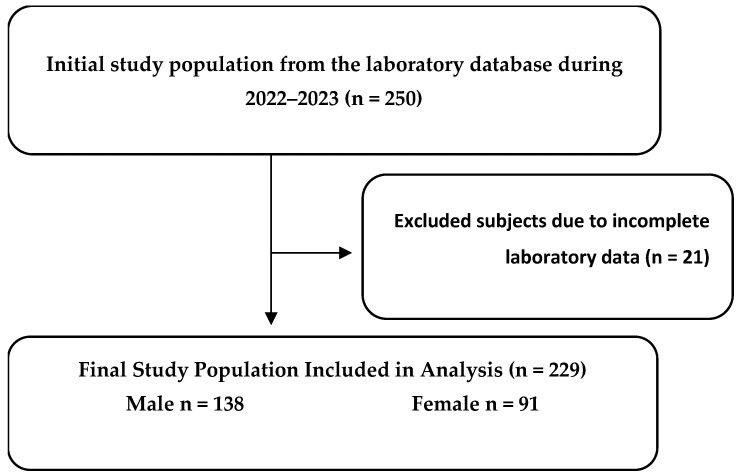
Flow diagram depicting the selection process of study participants.

Patients were eligible for inclusion if they had a documented diagnosis of chronic kidney disease (CKD) in the medical records of the nephrology clinic at King Saud Medical City and had simultaneously available hematological and biochemical laboratory tests, including renal function and lipid profile measurements (Figure 1). In routine clinical practice, CKD diagnosis is established by nephrologists in accordance with KDIGO 2024 guidelines, defined as abnormalities of kidney structure or function persisting for ≥3 months, including reduced estimated glomerular filtration rate (eGFR < 60 mL/min/1.73 m^2^) and/or markers of kidney damage (e.g., albuminuria) [26]. This study included non-dialysis CKD patients across KDIGO eGFR stages (G1–G5), with staging performed using the most recent available eGFR value at the time of data collection. Patients were excluded if they did not have a documented diagnosis of CKD, lacked complete hematological or biochemical laboratory data, were pregnant, were undergoing dialysis, or had end-stage renal disease requiring renal replacement therapy at the time of data collection.

### 2.3. Data Collection and Laboratory Parameters

The collected dataset included demographic characteristics, specifically age and sex, along with biochemical laboratory measurements obtained during routine clinical evaluation. Biochemical data comprised renal function parameters and a complete lipid panel. The lipid profile included measurements of triglycerides (TG), total cholesterol (TC), and high-density lipoprotein cholesterol (HDL-C). Low-density lipoprotein cholesterol (LDL-C) was calculated using the Friedewald formula (LDL-C = TC − HDL-C − TG/2.2, expressed in mmol/L). Non-high-density lipoprotein cholesterol (non-HDL-C) was calculated by subtracting HDL-C from TC (non-HDL-C = TC − HDL-C). Remnant cholesterol (RC) was indirectly estimated as TC − HDL-C − LDL-C. Given the use of the Friedewald equation, this calculation renders RC mathematically equivalent to TG/2.2; therefore, in this study, RC should be interpreted as a surrogate marker of triglyceride-rich lipoproteins (TRLs) rather than an entirely independent lipid fraction. Lipid abnormalities were defined based on established clinical lipid guidelines. Elevated total cholesterol was defined as TC ≥ 5.2 mmol/L, hypertriglyceridemia as TG ≥ 1.7 mmol/L, and elevated LDL-C as LDL-C ≥ 3.4 mmol/L. Low HDL-C was defined as ≤1.0 mmol/L in men or ≤1.2 mmol/L in women [27]. Elevated remnant cholesterol was defined as RC ≥ 0.6 mmol/L, while elevated non-HDL cholesterol was defined as ≥3.9 mmol/L [28,29].

### 2.4. CKD Classification

Patients were further stratified according to estimated glomerular filtration rate (eGFR) into CKD stages following the Kidney Disease: Improving Global Outcomes (KDIGO) 2024 Clinical Practice Guideline for the Evaluation and Management of Chronic Kidney Disease [26]. In addition to eGFR-based staging, metabolic complications were also evaluated. Uremia was defined as serum urea levels > 7.2 mmol/L, while hyperphosphatemia was defined as serum phosphate levels > 1.45 mmol/L, consistent with commonly accepted clinical laboratory reference ranges [30,31].

### 2.5. Statistical Analysis

All statistical analyses were performed using GraphPad Prism software (versions 9.0 and 10.5.0; GraphPad Software, San Diego, CA, USA). The distribution of continuous variables was assessed using the Shapiro–Wilk test to evaluate normality. As most variables showed non-normal distributions, continuous data were summarized as medians with interquartile ranges (IQR). Comparisons across more than two groups were conducted using the Kruskal–Wallis test. Where appropriate, post hoc pairwise comparisons were performed using Dunn’s test. Two-group comparisons were evaluated using the Mann–Whitney U test. To account for multiple comparisons, false discovery rate (FDR) correction using the Benjamini–Hochberg method was applied, and corresponding q-values are reported in the tables. To investigate the relationships between lipid parameters and renal function indices, multivariable linear regression analyses were performed using GraphPad Prism v10.5.0. The diagnostic performance of lipid parameters was evaluated using receiver operating characteristic (ROC) curve analysis. The area under the curve (AUC) was calculated to assess the ability of HDL-C and remnant cholesterol (RC) to discriminate between CKD stages and the presence of uremia. All statistical tests were two-tailed, and *p*-values < 0.05 were considered statistically significant.

## 3. Results

### 3.1. Descriptive Characteristics of the Included Subjects

Table 1 summarizes the baseline characteristics of the study subjects. The median age was 59 years (±46–69), with 60.3% males. The median BMI was 27.3 kg/m^2^ (IQR: 23.8–31.6). Renal function parameters reflected impaired kidney function, with a median eGFR of 37 mL/min/1.73 m^2^ (IQR: 21–61). The median blood urea nitrogen (BUN) was 9.6 mg/dL (IQR: 6.00–15.95), while serum creatinine levels had a median value of 158.9 µmol/L (IQR: 110.9–245.2). Regarding comorbidities, hypertension was highly prevalent (84.3%), and 44.1% of participants had type 2 diabetes, while 1.75% were smokers. Moreover, medication use among the study patients is summarized in Table 2.

### 3.2. Prevalence of Dyslipidemia Abnormalities in the Study Population

The overall lipid profile of the CKD patients included in this study is summarized in Table 3. Median lipid concentrations were 4.14 mmol/L (IQR 3.48–4.74) for total cholesterol, 1.17 mmol/L (0.92–1.79) for triglycerides, 2.52 mmol/L (1.90–3.13) for LDL-C, 1.04 mmol/L (0.87–1.24) for HDL-C, 0.47 mmol/L (0.32–0.69) for remnant cholesterol (RC), and 3.03 mmol/L (2.42–3.68) for non-HDL cholesterol. Notably, the median values of the overall CKD population remained largely within commonly accepted clinical reference ranges, despite a considerable proportion of patients exhibiting lipid abnormalities. Based on established thresholds, low HDL-C represented the most prevalent abnormality (49.78%), followed by elevated RC (31.44%) and hypertriglyceridemia (25.76%), whereas elevated TC, LDL-C, and non-HDL cholesterol were observed in 20.52%, 18.78%, and 20.09% of patients, respectively. Given that the overall medians did not markedly exceed clinical cutoffs, subsequent analyses stratified patients according to CKD stage, uremic status, and hyperphosphatemia to further explore lipid alterations across different CKD severity and metabolic states.

### 3.3. Distribution of Lipid Parameters Across eGFR-Defined CKD Stages

**Figure 2:**
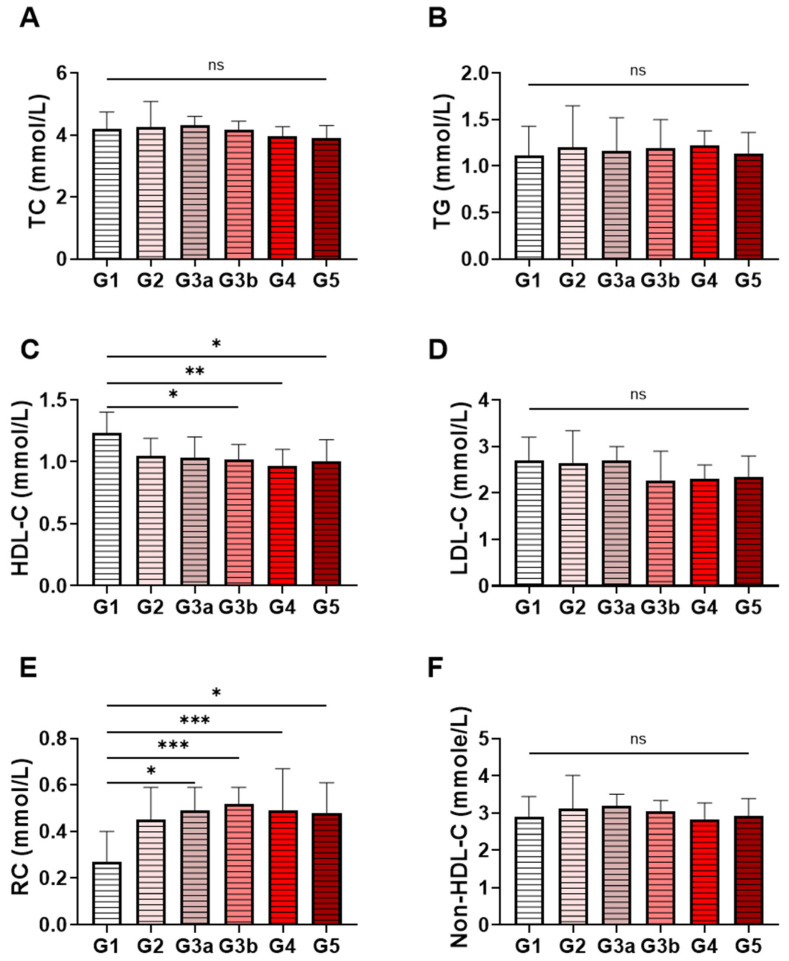
Lipid parameter distribution across eGFR categories in non-dialysis CKD patients. Bar charts show the median and interquartile range (IQR) levels of (**A**) total cholesterol (TC), (**B**) triglycerides (TG), (**C**) high-density lipoprotein cholesterol (HDL-C), (**D**) low-density lipoprotein cholesterol (LDL-C), (**E**) remnant cholesterol (RC), and (**F**) non-HDL-C across KDIGO-defined eGFR groups. Statistical significance is indicated as ns (not significant), * *p* < 0.05, ** *p* < 0.01 and *** *p* < 0.001.

Figure 2 illustrates the distribution of lipid parameters across different stages of CKD. In Figure 2A, Total cholesterol (TC) showed modest variation across groups, with higher medians in earlier eGFR categories (TC: 4.200–4.300 across G1–G3a) and lower medians in more advanced stages (TC: 3.955 in G4 and 3.900 in G5). Triglycerides (TG; Figure 2B) were broadly similar across strata (medians 1.110–1.220), while LDL-C and non–HDL-C (Figure 2D,F) were comparable in earlier categories (LDL-C; 2.650–2.700 across G1–G3a, non–HDL: 2.900–3.180 across G1–G3a) and lower in later categories (LDL-C; 2.270–2.350 across G3b–G5, non–HDL: 2.830 in G4 and 2.910 in G5).

Across eGFR categories (G1–G5), HDL-C (Figure 2C) showed a downward shift with lower eGFR, with a median HDL-C that was highest in G1 at 1.230 (IQR 1.06–1.47) and decreased across categories, reaching 0.965 (IQR 0.80–1.20) in G4, with a median of 1.000 (IQR 0.83–1.20) in G5. In parallel, remnant cholesterol, RC (Figure 2E), was lowest in G1 at 0.270 (±0.20–0.44) and remained higher from G2 onward, with medians ranging from 0.450 to 0.520 across G2–G5 and the highest median observed in G3b at 0.520 (±0.37–0.75).

### 3.4. Distribution of Lipid Parameters Between Non-Uremic and Uremic CKD Patients

**Figure 3:**
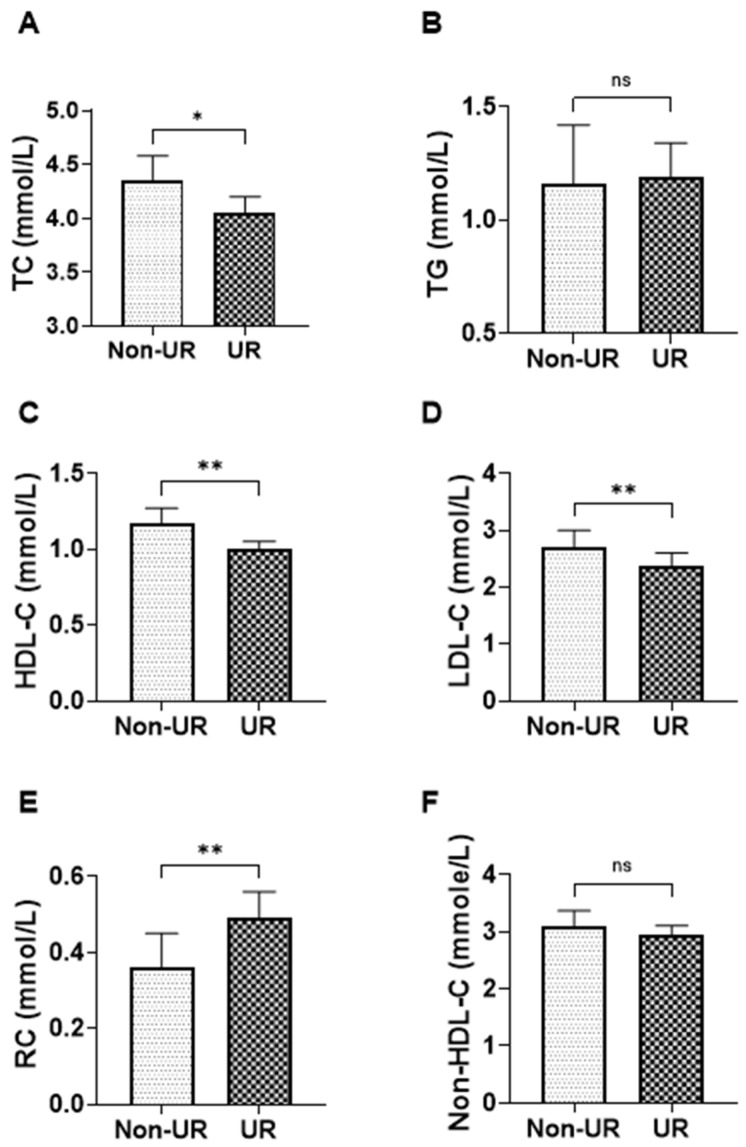
Lipid parameters according to uremic status in non-dialysis CKD patients. Bar charts show the median and interquartile range (IQR) levels of (**A**) total cholesterol (TC), (**B**) triglycerides (TG), (**C**) high-density lipoprotein cholesterol (HDL-C), (**D**) low-density lipoprotein cholesterol (LDL-C), (**E**) remnant cholesterol (RC), and (**F**) non-HDL-C according to uremic status in non-dialysis CKD patients. Statistical significance is indicated as ns (not significant), * *p* < 0.05 and ** *p* < 0.01.

Figure 3 illustrates the comparison of lipid parameters and selected atherogenic indices between non-uremic CKD patients (non-UR) and uremic CKD patients (UR). In comparisons by uremic status, HDL-C was lower in UR patients than in non-UR patients, with medians of 1.000 (IQR 0.80–1.19) versus 1.170 (0.97–1.39). Remnant cholesterol was higher in UR patients, with a median of 0.490 (0.37–0.71) compared with 0.360 (0.23–0.52) in the non-UR group, indicating a greater remnant lipoprotein burden in the uremic stratum.

Total cholesterol and LDL cholesterol were also lower in the UR group (TC: 4.050 [3.36–4.62]; LDL: 2.370 [1.78–3.00]) compared with the non-UR group (TC: 4.350 [3.70–5.46]; LDL: 2.700 [2.28–3.33]). Non–HDL cholesterol showed a smaller difference (2.950 [2.32–3.63] in the UR group vs. 3.105 [2.55–3.78] in the non-UR group). Triglycerides were similar between groups (1.190 [0.93–1.90] vs. 1.160 [0.91–1.70]).

### 3.5. Distribution of Lipid Parameters Among CKD Patients Stratified by Phosphate Levels

**Figure 4:**
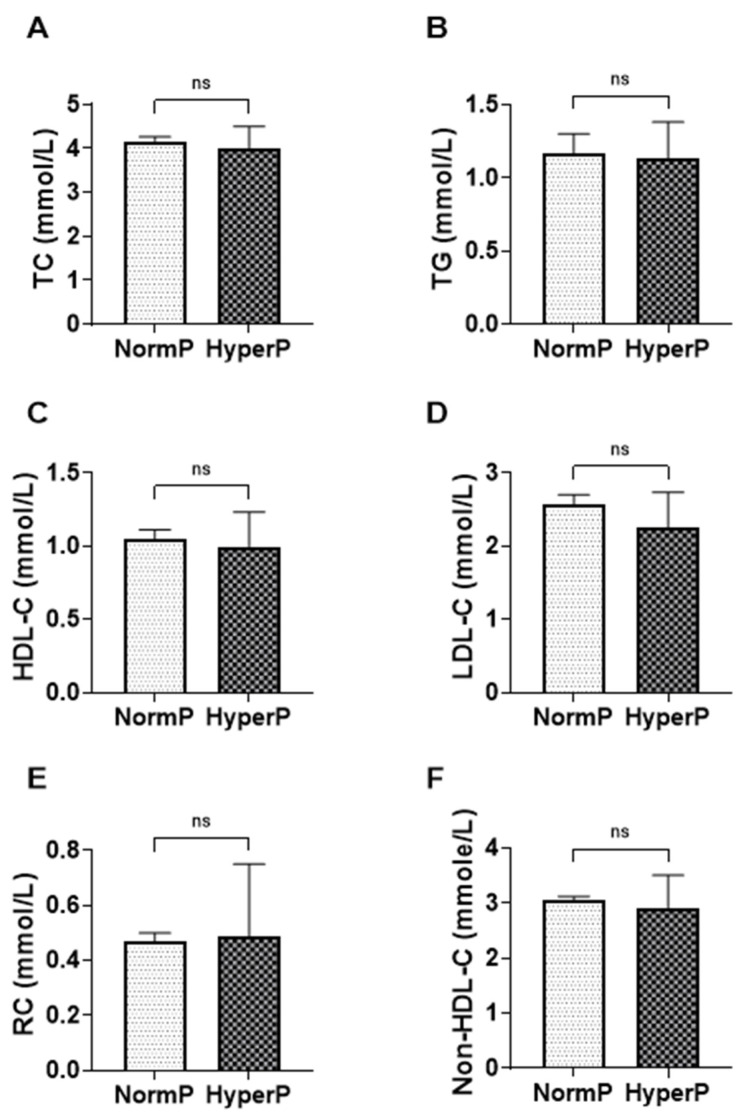
Lipid parameters according to phosphate status in non-dialysis CKD patients. Bar charts show the median and interquartile range (IQR) levels of (**A**) total cholesterol (TC), (**B**) triglycerides (TG), (**C**) high-density lipoprotein cholesterol (HDL-C), (**D**) low-density lipoprotein cholesterol (LDL-C), (**E**) remnant cholesterol (RC), and (**F**) non-HDL-C according to phosphate status in non-dialysis CKD patients. Statistical significance is indicated as ns (not significant).

When stratified by phosphate status, HDL cholesterol was similar across groups, with a median of 1.050 (±0.89–1.24) in the normal phosphate group and 0.995 (IQR 0.83–1.24) in the hyperphosphatemia group (Figure 4). Remnant cholesterol medians were also close between levels, at 0.470 (0.31–0.67) with normal phosphate and 0.485 (0.38–0.91) with hyperphosphatemia; however, the hyperphosphatemia group showed a wider interquartile range for RC.

Other lipid measures were broadly comparable. Total cholesterol was 4.170 (3.50–4.73) in the normal phosphate group and 4.015 (3.29–5.04) in the hyperphosphatemia group. LDL cholesterol was lower in hyperphosphatemia (2.250 [1.86–3.10]) compared with normal phosphate (2.570 [1.92–3.20]). Triglycerides were similar (1.170 [0.93–1.80] vs. 1.130 [0.88–1.56]), and non–HDL cholesterol showed modest differences (3.060 [2.37–3.65] vs. 2.915 [2.45–3.72]).

### 3.6. Multivariable Associations of HDL-C and RC with eGFR in CKD Patients

Table 4 presents multilinear regression analyses examining the associations between lipid parameters and eGFR (mL/min/1.73 m^2^) among patients with chronic kidney disease (CKD). In the unadjusted model (Model 1), HDL-C showed a significant positive association with eGFR (β = 59.55, 95% CI: 26.74–92.37, *p* = 0.0004), which remained significant after adjustment for age, sex, and BMI (Model 2) (β = 55.63, 95% CI: 25.62–85.65, *q* = 0.0006) and after further adjustment for smoking status, type 2 diabetes, and hypertension (Model 3) (β = 48.82, 95% CI: 19.27–78.37, *q* = 0.003).

Conversely, RC demonstrated a consistent inverse association with eGFR across all models. In Model 1, RC was significantly associated with lower eGFR (β = −27.02, 95% CI: −38.48 to −15.57, *q* < 0.0001). This relationship persisted in Model 2 (β = −19.81, 95% CI: −30.34 to −9.27, *q* = 0.0006) and Model 3 (β = −19.92, 95% CI: −30.13 to −9.70, *q* = 0.0005).

### 3.7. Multivariable Associations of HDL-C and RC with BUN Levels in CKD Patients

Table 5 presents multilinear regression analyses evaluating the relationship between lipid parameters and blood urea nitrogen (BUN, mg/dL) in patients with chronic kidney disease (CKD). HDL-C was inversely associated with BUN across all models. In the unadjusted model (Model 1), HDL-C showed a significant negative association with BUN (β = −15.28, 95% CI: −22.81 to −7.76, *q* < 0.0001). This relationship remained significant after adjustment for age, sex, and BMI (Model 2) (β = −16.43, 95% CI: −24.10 to −8.75, *q* = 0.0004) and after further adjustment for smoking status, type 2 diabetes, and hypertension (Model 3) (β = −14.33, 95% CI: −21.86 to −6.81, *q* = 0.0014).

Conversely, RC demonstrated a significant positive association with BUN. In Model 1, RC was associated with higher BUN levels (β = 3.46, 95% CI: 0.73–6.19, *q* = 0.013), and this association remained significant in Model 2 (β = 3.33, 95% CI: 0.56–6.09, *q* = 0.0374) and Model 3 (β = 3.37, 95% CI: 0.70–6.04, *q* = 0.0315).

### 3.8. ROC Analysis of HDL-C and Remnant Cholesterol for Identifying CKD Stages and Metabolic Complications

Receiver operating characteristic (ROC) analysis was performed to evaluate the discriminatory ability of HDL-C and remnant cholesterol (RC) across CKD stages and selected metabolic complications (Table 6). Both lipid parameters demonstrated moderate diagnostic performance across CKD stages, with AUC values ranging from 0.65 to 0.71 for HDL-C and 0.68 to 0.78 for RC. Notably, RC showed progressively higher discriminatory capacity with advancing CKD severity, reaching the highest AUC in CKD stage G4 (AUC = 0.78, 95% CI 0.67–0.89, *p* < 0.0001), whereas HDL-C showed relatively stable but slightly lower AUC values across stages.

For the detection of uremia, both HDL-C and RC demonstrated modest but significant discriminatory performance, with AUC values of 0.66 (95% CI 0.58–0.74, *p* < 0.001) and 0.65 (95% CI 0.57–0.74, *p* < 0.001), respectively. In contrast, neither HDL-C nor RC showed significant discriminatory ability for hyperphosphatemia, with AUC values close to 0.5 and non-significant *p*-values.

## 4. Discussion

In this retrospective analysis of CKD patients stratified by KDIGO eGFR stages, uremic status, and phosphate status, several important observations emerged. First, declining eGFR was associated with a consistent increase in RC and reduction in HDL-C, whereas TC, LDL-C, TG, and non–HDL-C remained largely unchanged across CKD stages. Second, classification by phosphate status did not reveal significant differences in any lipid parameters. Third, uremic status was associated with reduced HDL-C and increased RC, along with lower TC and LDL-C, while TG and non–HDL-C did not differ significantly. Together, these findings suggest that lipid abnormalities in CKD are characterized more by qualitative shifts in lipoprotein composition, particularly involving HDL and triglyceride-rich lipoproteins, rather than a uniform increase in total atherogenic cholesterol burden.

In the present study, remnant cholesterol (RC) increased with declining eGFR and was higher in uremic patients. This observation aligns with evidence from large population-based cohorts linking elevated RC to impaired renal function. The Copenhagen General Population Study demonstrated that reduced eGFR is associated with higher RC levels, and that increasing RC is linked to a greater risk of myocardial infarction and atherosclerotic cardiovascular disease [32]. Consistent findings have been reported in diabetic CKD, where RC independently predicts renal function decline [33], and in meta-analytic data showing an association between elevated RC, CKD risk, and lower eGFR [34]. Collectively, these studies highlight remnant cholesterol (RC) as an important lipid marker in individuals with kidney dysfunction. Given that RC was indirectly calculated using the Friedewald-based approach in this dataset, it reflects the cholesterol content of triglyceride-rich lipoprotein (TRL) remnants, including chylomicron remnants, very-low-density lipoproteins (VLDL), and intermediate-density lipoproteins (IDL). These remnant particles are increasingly recognized as highly atherogenic owing to their capacity to infiltrate the arterial wall, impair endothelial function, and promote vascular inflammation.

Several mechanisms may explain the elevation of RC observed in patients with CKD. Accumulation of RC is mainly due to increased hepatic production of triglyceride-rich lipoproteins, together with impaired peripheral clearance of remnant particles [35]. Elevation in hepatic lipoprotein secretions may be driven by insulin resistance and hyperinsulinemia, metabolic disturbances, which are often present in CKD patients [36]. In addition, experimental studies suggest that impaired hepatic clearance may further exacerbate remnant accumulation. Animal models of chronic renal failure have demonstrated down-regulation of hepatic receptors involved in remnant uptake, particularly the LDL receptor–related protein (LRP), which mediates the clearance of IDL and chylomicron remnants [37]. Others have shown that adipose tissue LPL activity is diminished in CKD patients, leading to accumulation of chylomicron remnants and IDL [38,39]. Collectively, these alterations highlight the complex disturbances in lipoprotein metabolism that underline the characteristic remnant-rich dyslipidemia observed in CKD patients.

In the present study, HDL-C levels progressively declined with decreasing eGFR among non-dialysis CKD patients, indicating a close relationship between impaired renal function and reduced HDL concentrations, which is consistent with findings from previous studies. Moreover, the KNOW-CKD study demonstrated an interesting relationship between HDL-C levels and CKD outcomes. In this large prospective cohort of more than 2000 CKD patients, both very low HDL-C (<30 mg/dL) and extremely high HDL-C levels were associated with a significantly higher risk of CKD progression and adverse renal outcomes, suggesting a complex relationship between HDL metabolism and kidney disease progression [40]. Mechanistically, CKD is associated with reduced apolipoprotein A-I synthesis, impaired activity of enzymes involved in HDL maturation such as lecithin-cholesterol acyltransferase (LCAT) [41]. Additionally, it was shown that a decline in renal function assessed by eGFR induces unfavorable changes in HDL composition and impairs its ability to promote VLDL lipolysis [42], highlighting the development of dysfunctional HDL particles in renal disease.

The stability of TC, LDL-C, and non–HDL-C across CKD stages is particularly informative. Despite their established roles as markers of cardiovascular risk, these parameters may undergo qualitative rather than quantitative alterations in CKD. Lipidomic studies support this concept, showing that while total LDL lipid and cholesterol content may remain comparable to non-CKD controls, the composition of lipid subclasses is altered, including reductions in phosphatidylcholines, sulfatides, and ceramides, alongside increased N-acyltaurines [43]. In this context, the observed rise in remnant cholesterol (RC) likely reflects a redistribution of cholesterol within the existing atherogenic lipoprotein pool toward triglyceride-rich remnant particles. This distinction is clinically important, as conventional lipid measures may fail to capture these compositional changes that contribute to cardiovascular risk in CKD. Accordingly, incorporating RC alongside traditional lipid markers may improve risk stratification, although longitudinal studies with detailed lipoprotein profiling are needed to clarify the prognostic significance of these alterations.

Uremia, characterized by the accumulation of metabolic waste due to impaired renal clearance, is a hallmark of advanced kidney dysfunction and, if untreated, constitutes a life-threatening condition [44]. Our findings showed that uremic status was associated with lower HDL-C and elevated RC, suggesting that uremia may preferentially disrupt lipoprotein metabolism rather than broadly alter total lipid concentrations. Uremic toxin accumulation, chronic inflammation, and protein-energy wasting are known metabolic disturbances in CKD that can impair HDL metabolism and promote triglyceride-rich lipoprotein abnormalities [24,45]. In contrast, phosphate status did not discriminate against lipid profiles in this cohort. Although hyperphosphatemia is a key feature of CKD-mineral and bone disorder and an established contributor to cardiovascular risk [46], its pathogenic effects are likely mediated through mechanisms distinct from classical dyslipidemia, including vascular smooth muscle cell osteogenic transformation, arterial stiffness, and vascular calcification [47,48]. The association of elevated RC and reduced HDL-C with uremia suggests that these lipid measures may function as surrogate indicators of uremic metabolic burden in non-dialysis CKD. Their alteration may identify patients with more advanced biochemical disturbance, even before overt kidney failure or dialysis dependence develops.

ROC curve analyses demonstrated modest discriminatory performance of lipid parameters and related indices in identifying CKD-related stratifications, with AUC values indicating limited classification accuracy. Importantly, the observed discriminatory performance may be enhanced when these markers are incorporated into multivariable models that include additional clinical, metabolic, and inflammatory factors not available in the present dataset. In this context, lipid parameters—particularly RC and HDL-C—may have greater utility as supportive or complementary markers within integrated risk prediction approaches, rather than as independent discriminators. The modest AUC values likely reflect the complex and multifactorial nature of CKD-associated dyslipidemia, where lipid alterations coexist with diverse metabolic and renal-specific mechanisms.

This study benefits from the use of fasting lipid measurements, consistent calculation of LDL-C, and comprehensive adjustment for relevant clinical covariates, including age, sex, BMI, smoking, type 2 diabetes, and hypertension. However, several limitations should be acknowledged. The retrospective and cross-sectional design precludes causal inference. Lipid functionality, particularly HDL efflux capacity, and detailed lipoprotein profiling were not assessed. Additionally, the lack of longitudinal follow-up limits the ability to evaluate associations with cardiovascular outcomes. Moreover, detailed dietary information was not available, despite its potential influence on lipid metabolism and its modification across different stages of CKD and treatment strategies. Furthermore, key metabolic and atherogenic markers such as serum uric acid and lipoprotein(a) were not available in this dataset; future studies incorporating these parameters may provide a more comprehensive assessment of cardiometabolic risk in non-dialysis CKD populations.

## 5. Conclusions

In conclusion, this study demonstrates that CKD severity is associated with a distinct lipid pattern characterized by reduced HDL-C and increased RC, in the absence of changes in non–HDL-C or traditional lipid parameters. Uremia appears to selectively influence HDL-C and RC levels, while phosphate status does not meaningfully affect lipid profiles. These findings highlight the importance of qualitative lipoprotein alterations in CKD and support the potential role of RC and HDL-C as sensitive markers of CKD-related dyslipidemia and uremia.

## Figures and Tables

**Table 1:** Baseline Characteristics of the Study Cohort.

Variable	Median (IQR) or *n* (%)
Age	59 (46–69)
Male	138 (60.26%)
BMI	27.3 (23.8–31.6)
eGFR	37 (21–61)
BUN	9.6 (6.00–15.95)
Creatinine	158.9 (110.9–245.2)
% HTN	193 (84.3%)
% T2D	101 (44.1%)
% Smoker	4 (1.75%)

Continuous variables are presented as median (IQR), and categorical variables as *n* (%). Abbreviations: BMI, body mass index; eGFR, estimated glomerular filtration rate; BUN, blood urea nitrogen; HTN, hypertension; T2D, type 2 diabetes; IQR, interquartile range.

**Table 2:** Distribution of Major Medication Classes in the Study Subjects.

Medication Class	n	%
Antihypertensive agents	193	84.3%
Lipid-lowering therapy	128	55.9%
Atorvastatin	89	38.9%
Rosuvastatin	39	17.0%
Antidiabetic therapy	98	42.8%
Diuretics	97	42.3%
CKD-related metabolic therapy	85	37.1%
Vitamin/mineral supplementation	120	52.4%
Iron/hematologic therapy	90	39.3%
Antiplatelet/anticoagulant therapy	64	27.9%
Gastroprotective therapy	102	44.5%
Immunosuppressive/anti-inflammatory drugs	63	27.5%

**Table 3:** Lipid Profile and Prevalence of Dyslipidemia Abnormalities in the overall CKD patients.

Lipid Parameter	Clinical Threshold (mmol/L)	Median (IQR), mmol/L	Abnormal *n* (%)
Total cholesterol (TC)	≥5.2	4.14 (3.48–4.74)	47 (20.52%)
Triglycerides (TG)	≥1.7	1.17 (0.92–1.79)	59 (25.76%)
LDL cholesterol (LDL-C)	≥3.4	2.52 (1.90–3.13)	43 (18.78%)
HDL cholesterol (HDL-C)	≤1.0 (men)/≤1.2 (women)	1.04 (0.87–1.24)	114 (49.78%)
Remnant cholesterol (RC)	≥0.6	0.47 (0.32–0.69)	72 (31.44%)
Non-HDL cholesterol	≥3.9	3.03 (2.42–3.68)	46 (20.09%)

Abbreviations: TC, total cholesterol; TG, triglycerides; LDL-C, low-density lipoprotein cholesterol; HDL-C, high-density lipoprotein cholesterol; RC, remnant cholesterol; IQR, interquartile range.

**Table 4:** Multivariable Regression Analysis of eGFR with HDL-C and Remnant Cholesterol.

Variable	Model 1 β (95% CI)	*q* Value	Model 2 β (95% CI)	*q* Value	Model 3 β (95% CI)	*q* Value
HDL-C	59.6 (26.7 to 92.4)	0.0004	55.6 (25.6 to 85.7)	0.0006	48.8 (19.3 to 78.4)	0.003
R^2^	0.053		0.285		0.340	
RC	−27.0 (−38.5 to −15.6)	<0.0001	−19.8 (−30.3 to −9.3)	0.0006	−19.9 (−30.1 to −9.7)	0.0005
R^2^	0.087		0.286		0.351	

Abbreviations: eGFR, estimated glomerular filtration rate; HDL-C, high-density lipoprotein cholesterol; RC, remnant cholesterol. Model 1: Unadjusted model; Model 2: Adjusted for age, sex, and BMI; Model 3: Adjusted for age, sex, BMI, type 2 diabetes, hypertension, and smoking.

**Table 5:** Multivariable Regression Analysis of BUN with HDL-C and Remnant Cholesterol.

Variable	Model 1 β (95% CI)	*q* Value	Model 2 β (95% CI)	*q* Value	Model 3 β (95% CI)	*q* Value
HDL-C	−15.3 (−22.8 to −7.8)	<0.0001	−16.4 (−24.1 to −8.8)	0.0004	−14.3 (−21.9 to −6.8)	0.0014
R^2^	0.066		0.122		0.181	
RC	3.5 (0.7–6.2)	0.013	3.3 (0.6–6.1)	0.0374	3.4 (0.7–6.0)	0.0315
R^2^	0.027		0.076		0.153	

Abbreviations: BUN, blood urea nitrogen; HDL-C, high-density lipoprotein cholesterol; RC, remnant cholesterol. Model 1: Unadjusted model; Model 2: Adjusted for age, sex and BMI; Model 3: Adjusted for age, sex, BMI, type 2 diabetes, hypertension, and smoking.

**Table 6:** Discriminatory performance of HDL-C and RC for CKD stages and complications.

	HDL-C (AUC, 95% CI)	*p* Value	RC (AUC, 95% CI)	*p* Value
G2	0.66 (0.52 to 0.80)	0.0297	0.68 (0.54 to 0.82)	0.0169
G3a	0.65 (0.51 to 0.79)	0.0375	0.71 (0.58 to 0.84)	0.0034
G3b	0.68 (0.56 to 0.81)	0.0069	0.75 (0.64 to 0.87)	0.0002
G4	0.71 (0.60 to 0.83)	0.0013	0.78 (0.67 to 0.89)	<0.0001
G5	0.71 (0.58 to 0.85)	0.0051	0.71 (0.58 to 0.85)	0.0052
Uremia	0.66 (0.58 to 0.74)	<0.001	0.65 (0.57 to 0.74)	<0.001
Hyperphosphatemia	0.58 (0.48 to 0.68)	0.1339	0.53 (0.43 to 0.64)	0.4916

Abbreviations: AUC, area under the receiver operating characteristic curve; CI, confidence interval; HDL-C, high-density lipoprotein cholesterol; RC, remnant cholesterol; CKD, chronic kidney disease; G2–G5, CKD stages based on eGFR classification.

## Data Availability

The datasets used in this study can be obtained from the corresponding author (Y.A.) upon reasonable request and with the approval of King Saud Medical City.
